# Prevalence, characteristics, and associated risk factors of drug consumption and chemsex use among individuals attending an STI clinic (EpITs STUDY)

**DOI:** 10.3389/fpubh.2023.1285057

**Published:** 2023-10-31

**Authors:** Marta Rosas Cancio-Suárez, Raquel Ron, Jorge Díaz-Álvarez, Javier Martínez-Sanz, Sergio Serrano-Villar, Santiago Moreno, Matilde Sánchez-Conde

**Affiliations:** ^1^Department of Infectious Diseases, Hospital Ramón y Cajal, Carretera de Colmenar, Madrid, Spain; ^2^Ramón y Cajal Research Institute (IRYCIS), Madrid, Spain; ^3^CIBER de Enfermedades Infecciosas (CIBERINFEC), Madrid, Spain; ^4^Department of Medicine, University of Alcalá de Henares, Madrid, Spain

**Keywords:** sexually transmitted infections, chemsex, drug use, prevalence, risk factors, interventions, sexual health

## Abstract

Sex-related drug consumption and its health-related consequences have gained relevance in the assessment of patients with sexually transmitted infections (STIs), which pose a significant challenge to public health. We aim to assess the prevalence and characteristics of drug consumption and chemsex practices, describe the associated risk factors among general individuals attending an STI clinic, and evaluate the psychological impact associated with these behaviors. We conducted an online anonymous survey offered to patients with a diagnosis of STI in a tertiary hospital in Spain. Data included sociodemographic characteristics, sexual preferences and behavior, and assessment of drug use, chemsex, and psychological and mental health symptoms. Data from 145 subjects was collected, with a higher proportion of cis-gender men (71%), and a median age of 32 years. 64 participants (44%) reported drug use in the last year, with an observed 33.8% prevalence of chemsex consumption. Drug use and chemsex were more frequent among cis-gender men, Men who have Sex with Men (MSM), people living with HIV (PLHIV), and those reporting previous group sex. Poppers and cannabis were the most frequently reported drugs, with a prevalence close to 20% for cocaine, mephedrone, extasis, and GHB. Consequences related to drug use included unpleasant physical sensations, sexual dysfunction, and impaired sexual experience after reduction or drug discontinuation. The prevalence of drug use and chemsex practices are high among patients evaluated for STIs, especially between men, MSM, and subjects practicing group sex. The study highlights the urgent need for targeted interventions on prevention and reduction of their impact on health and social well-being.

## 1. Introduction

Sexually Transmitted Infections (STIs) are a major health problem worldwide that cause genital and reproductive morbidity. In 2020, WHO estimated 374 million new infections, with transmission rates progressively increasing (1). Vulnerable groups for STIs acquisition include sex workers, men who have sex with men (MSM), people who inject drugs (PWID), prison inmates, and adolescents in countries with high HIV burden (2).

In addition, the use of recreational drugs in a sexual context, commonly known as chemsex, has gained special relevance during the last decade, with a high prevalence described in some populations, such as MSM or People living with HIV (PLHIV) (3), outlining the need for harm reduction interventions and preventive actions (4). While a wide range of drugs are involved in chemsex, substances like gamma hydroxybutyrate (GHB/GBL), cocaine, mephedrone, and methamphetamine are commonly reported (3, 5, 6). The use of chemsex has been related to several harmful consequences. Recently published data found a higher risk of psychosis development, added to other risk factors such as foreign or ethnic minority status, location in large cities, stress and anxiety, trauma, loneliness, and STIs (7). Notably, in studies conducted among MSM practicing chemsex, a significantly higher association with STIs has been observed compared to those who do not engage in sex-related drug consumption. Risk increased by as much as 2.5-fold (8).

Social networks are other significant contributing factors in high-risk behaviors (6, 9), and have been previously related to chemsex use between MSM (10). Frequently, the increase in risky sexual practices facilitated by mobile apps coincides with the use of chemsex, as these apps enable encounters in settings conducive to recreational drug use.

Although the chemsex phenomenon has been previously studied among specific groups, like MSM or PLHIV in urban settings (6, 10, 11), its prevalence in different populations or contexts is not well known, and there is a lack of data about drug consumption and chemsex between heterosexual couples or women. Increasing knowledge about drug consumption and chemsex, the behavioral patterns related, and their possible implications on public health are the main issues to be addressed by public policies, given its possible consequences on STIs incidence, mental health, and social well-being (12, 13).

Here, we aim to assess the prevalence and characteristics of drug consumption and chemsex practices, describe the associated risk factors among general individuals attending an STI clinic, and evaluate the psychological impact associated with these behaviors.

## 2. Methods

### 2.1. Inclusion criteria and collected data

We conducted an anonymous online self-administered survey among volunteer participants from a general STI clinic in a tertiary hospital in Madrid, Spain. Patients are referred to our clinic from different departments, including Emergency, Primary Care, and Infectious Diseases. Participation was voluntary with no incentive of any kind. Inclusion criteria were patients over 18 years of age, with a confirmed diagnosis of STI, regardless of gender, sexual preferences or type of STI diagnosed. After a detailed explanation of the purpose and methods of the study, written informed consent was collected from those who agreed to participate. Doctors provided participants with a card containing a QR code for direct access to an online survey, and the results were automatically anonymized and recorded in the REDcap ^®^8 database. Patient recruitment was prospectively carried out from January to December 2022. The questionnaire comprised 26 main items. Data collected included sociodemographic characteristics, medical history including previous STI and HIV status, sexual preferences, sexual risk behaviors, use of social apps, and the Hospital Anxiety and Depression Scale (HADS) score. This scale is a screening tool with 14 items evaluating the risk of anxiety and depression, with a total score between 0-21, considering normal values (0-7), possible anxiety or depression case (8-10), and confirmed case (11-21) (14). Evaluation of drugs and chemsex consumption included type of substance, frequency and location of use, route of administration, sex-related use, and possible consequences derived from consumption. Chemsex was defined by an affirmative answer to the use of any of the following drugs in the sexual context: Methamphetamine, GHB/GBL, Mephedrone, Cocaine, Ketamine, Poppers, Speed, and others (describing which type). No data involving personal identification were collected. Physical and psychological consequences related to drug use were collected by a predefined options list. The complete list of recorded variables is included in the Supplementary material 1. The study was conducted following the Declaration of Helsinki and the protocol was approved by the ethics committee of Ramón y Cajal Hospital. The primary endpoint of the study was to evaluate the prevalence and characteristics of drug use and chemsex. Secondary endpoints include the evaluation of the risk factors associated with drug consumption and chemsex, and its consequences on mental health and well-being.

### 2.2. Statistical analysis

Data are presented using absolute and relative frequencies for categorical variables, and median and interquartile range (IQR) for continuous variables. We calculated the prevalence of drug consumption and chemsex use for the overall participants and compared sociodemographic and sexual-related characteristics based on drug consumption and chemsex-reported use, using the t-test for continuous variables and the chi-square test for categorical variables. We conducted a logistic regression analysis, with drug use as the dependent variable, to evaluate the association between different sociodemographic characteristics, sexual-related factors, medical history, and drug consumption. Variables with a significant association in the univariate analysis (*p* < 0.05) were then included in the multivariable model following a backward stepwise strategy. We applied the same methods for the evaluation of possible risk factors related to chemsex use. All analyses were performed using Stata (StataCorp. 2019. 16.1. Statistical Software; StataCorp LLC, College Station, TX, USA).

## 3. Results

We collected data from 145 subjects, including 103 cis-gender men and 31 cis-gender women, and a median age of 32 years (IQR 26–42). A total of 96 participants (66%) reported previous or current drug consumption, with 67% (*n* = 64) reporting consumption during the last year. In the drug consumption group, 51% (*n* = 49) reported substance use in the context of sexual relationships, representing a total prevalence of chemsex among the study subjects of 33.8%.

Sociodemographic characteristics and sexual behaviors according to the history of drug consumption are described in Table 1. Subjects in the drug use group were more frequently cis-men, reported MSM, with high educational level and history of group sex in the last year (>3 partners in the same intercouse). Moreover, there was a higher proportion of PLHIV in the consumption group (27%). Chemsex use was more prevalent among men, representing 96% of all the users, and notably in the group of MSM [84% versus 6% of Men Who Have Sex with Women (MSW) or bisexual men]. The prevalence was also higher between PLHIV (43%), subjects reporting no stable couple (71%), with a total number of sexual partners of 12 or more in the last year, and engaging in group sex (63%) (Supplementary material 2).

**Table 1:** Sociodemographic and sexual related characteristics by drug use.

	No consumption *n* = 49	Consumption = 96
Age	32 (20–42)	36 (25–47)
Genre		
Man cis	23 (47%)	80 (83%)
Woman cis	20 (41%)	11 (11%)
Man trans	1 (2%)	2 (2%)
Not identified with previous	2 (4%)	1 (1%)
Birth place		
Spain	28 (57%)	61 (64%)
Europe	2 (4%)	1 (1%)
South America	14 (29%)	28 (29%)
North America	1 (2%)	0 (0%)
Centre America	2 (4%)	6 (6%)
Education level		
Primary studies not completed	0 (0%)	2 (2%)
Primary studies completed	1 (2%)	3 (3%)
Secondary	24 (49%)	36 (38%)
University	22 (45%)	54 (56%)
Income level		
None	8 (16%)	8 (8%)
<500 euros per month	3 (6%)	8 (8%)
500–1000 euros per month	6 (12%)	18 (19%)
1000–2000 euros per month	20 (41%)	46 (48%)
>2000 euros per month	11 (22%)	15 (16%)
HIV	4 (8%)	26 (27%)
Previous PreP use	4 (8%)	10 (10%)
Previous STI (last year)	22 (45%)	54 (56%)
Stable partner	23 (47%)	42 (44%)
Sexual partners in the last year		
1	17 (35%)	17 (18%)
2–4	16 (33%)	32 (33%)
6–12	5 (10%)	15 (16%)
12–24	3 (6%)	12 (12%)
24–36	1 (2%)	8 (8%)
>36	3 (6%)	10 (10%)
Groupal sex in the last year (>3)	1 (2%)	15 (16%)
Condom use		
>50% intercouse	28 (57%)	52 (54%)
<50% intercourse	16 (33%)	39 (41%)
Sexual partner		
MSM	19 (39%)	60 (62%)
MSW	5 (10%)	17 (18%)
WSM	18 (37%)	9 (9%)
WSW	1 (2%)	0 (0%)
Bisexual Men	0 (0%)	4 (4%)
Bisexual Women	1 (2%)	2 (2%)
Sexual services provided	2 (4%)	5 (5%)
Sexual services hired	2 (4%)	5 (5%)
Anxiety scale	8.49 (4.31–12.67)	8.00 (4.00–11.00)
Depression scale	5.04 (1.37–8.71)	4.92 (1.23–8.61)

Data are presented as mean (SD) for continuous measures, and n (%) for categorical measures.

Prep, preexposure prophylaxis; STI, sexually transmitted infections; MSM, men who have sex with men; MSW, men who have sex with women; WSM, women who have sex with men; WSW, women who have sex with women.

Characteristics of drug consumption are detailed in Table 2. The most frequently used substance in the last year was poppers (50%), followed by cannabis (41%), with 90% of popper users reporting consumption in the sexual context (data for cannabis not available). The prevalence of cocaine, mephedrone, extasis, and GHB use were similar, around 20%, and their sex-related consumption was 70, 90, 29, and 95%, respectively. Fifteen participants (15%) reported combined substance use. The most common place of use was home (43%), followed by sexual venues (15%). The most frequently consumed drugs in the MSM group were poppers, with a higher consumption of synthetic drugs, including methamphetamines, GHB, and mephedrone, in comparison to heterosexual couples. Consumption among MSW was more prominent for cocaine (23%) and alcohol (50%). Among drug users, 61% reported the use of dating apps in the last year. The target population of the most frequent apps reported were gay, bisexual, and transgender collectives (47%). The median scores for the HADS scale between groups for anxiety or depression assessment showed a moderate risk of anxiety, but we did not find significant differences between drug or chemsex users.

**Table 2:** Characteristics of drug consumption and by type of sexual partner.

	Drug users *N* = 96	MSM *n* = 83	MSW *n* = 26	WSM *n* = 30
Drug use with sexual partner	19 (20%)	17 (20%)	2 (8%)	0 (0%)
Groupal sex > 3		15 (18%)	1 (4%)	0 (0%)
Combined drug use	14 (15%)			
Type of drug				
Cannabis				
Last year	39 (41%)	28 (34%)	8 (31%)	5 (17%)
Cocaine				
Last year	20 (21%)	15 (18%)	6 (23%)	0 (0%)
Sex related use	14 (15%)	11 (13%)	3 (12%)	
Mephedrone				
Last year	20 (21%)	19 (23%)	1 (4%)	0 (0%)
Sex related use	18 (19%)	17 (20%)	1 (4%)	
Extasis				
Last year	17 (18%)	15 (18%)	3 (11%)	1 (3%)
Sex related use	5 (5%)	5 (6%)	1 (4%)	
Ketamine				
Last year	8 (8%)	8 (10%)	1 (4%)	0 (0%)
Sex related use	3 (3%)	3 (4%)	0 (0%)	
Anphetamine				
Last year	12 (12%)	11 (13%)	0 (0%)	0 (0%)
Sex related use	10 (10%)	9 (11%)		
GHB/GBL				
Last year	19 (20%)	18 (22%)	0 (0%)	0 (0%)
Sex related use	18 (19%)	17 (20%)		
Speed				
Last year	10 (10%)	9 (11%)	0 (0%)	0 (0%)
Sex related use	4 (4%)	4 (5%)		
Poppers				
Last year	48 (50%)	43 (52%)	6 (23%)	0 (0%)
Sex related use	43 (45%)	40 (48%)	4 (15%)	
Injected drug use	3 (3%)			
Sharing injection material	2 (2%)			
Smoke	38 (40%)	25 (30%)	8 (31%)	7 (32%)
Alcohol (≥1–2 times/week)	40 (42%)	28 (34%)	13 (50%)	9 (30%)
Viagra	29 (30%)	24 (29%)	5 (20%)	0 (0%)
Steroids	2 (2%)	3 (4%)	0 (0%)	0 (0%)
Proteic supplements	30 (31%)	28 (34%)	9 (35%)	2 (7%)
Ansiolytic drugs	29 (30%)	22 (27%)	2 (8%)	10 (33%)
Drug use location				
Home	41 (43%)			
Bar	7 (7%)			
Sexual venues	14 (15%)			
Cruising	7 (7%)			
Street	1 (1%)			
Drug use duration				
<4 h	27 (28%)			
4–12 h	16 (17%)			
12–24 h	5 (5%)			
24–48 h	1 (1%)			
Drug use expenses				
1–100 euros	25 (26%)			
101–500 euros	5 (5%)			
501–1000 euros	2 (2%)			

MSM, men who have sex with men; MSW, men who have sex with women; WSM, women who have sex with men; WSW, women who have sex with women.

Univariate analysis showed a significantly higher risk of drug consumption associated with cis-gender men and PLHIV, with an OR 6.32; 95%CI (2.65–15.1) and OR 3.99; 95%CI (1.30–12.2) respectively. Alcohol [OR 2.44; 95%CI (1.11–5.37)] and especially tobacco abuse [OR 7.21; 95%CI (2.39–21.70)] were also associated with a higher risk. Considering sexual-related factors, we found an association between previous history of STI and group sex as risk factors for drug consumption. Results are detailed in Table 3. Specific chemsex univariate analysis also showed a statistical association between PLHIV, smoking, previous STIs, and group sex as risk factors. Notably, we found a higher risk of chemsex use between MSM [OR 6.83 95% CI (1.87–24.95)] and a protective effect of having a stable partner. In the multivariate analysis, male sex [OR 5.70; 95%CI (2.03–16.02)], smoking [OR 9.20; 95%CI (2.66–31.79)], and group sex [OR 3.68; 95%CI (1.21–11.22)] showed a statistically significant association with drug use in the final model. For chemsex use, smoking, PLHIV and group sex remained statistically significant. Finally, different consequences associated with substance use were evaluated. Unpleasant physical sensations (14%), sexual dysfunction (10%), and reduced sexual pleasure (9%) after drug discontinuation were the most frequent experiences reported, with 7% of patients identifying panic attacks or anxiety and 7% work interference (Supplementary material 3).

**Table 3:** Risk factors associated with drug consumption and chemsex use.

Total drug use	Univariate analysis		Multivariate analysis Max model		Multivariate analysis Optimized model	
	OR (95% CI)	*p* value	OR (95% CI)	*p* value	OR (95% CI)	*p* value
Man cis (ref. woman cis)	6.32 (2.65–15.1)	<0.05	5.88 (0.30–113.59)	0.241	5.70 (2.03–16.02)	<0.05
HIV	3.99 (1.30–12.2)	<0.05	3.85 (0.72–20.71)	0.115	–	
Smoke	7.21 (2.39–21.70)	<0.05	10.50 (2.77–39.74)	0.001	9.20 (2.66–31.79)	<0.05
Alcohol (≥1–2 times/week)	2.44 (1.11–5.37)	<0.05	2.93 (1.04–8.22)	0.041	2.48 (0.97–6.32)	0.06
STI history	2.49 (1.16–5.32)	<0.05	2.41 (0.80–7.25)	0.119	–	
Group sex on the last year (≥3 people)	4.3 (1.66–11.1)	<0.05	3.68 (1.21–11.22)	0.022	3.68 (1.21–11.22)	<0.05
MSM (ref. MSW)	0.93 (0.30–2.85)	0.90	0.50 (0.12–1.98)	0.313	–	
WSM	0.15 (0.04–0.53)	<0.05	0.79 (0.04–14.08)	0.875	–	
Pay for sexual services	1.28 (0.24–6.84)	0.77				
Provide sexual services	1.26 (0.24–6.77)	0.78				
Stable partner	0.85 (0.42–1.69)	0.64				
Primary educational level (ref.university)	1.22 (0.12–12.40)	0.37				
Secondary educational level (ref.university)	0.61 (0.30–1.25)	0.37				
Condom use	0.76 (0.36–1.60)	0.47				
Anxiety scale	0.97 (0.90–1.05)	0.52				
Depression scale	0.99 (0.90–1.09)	0.86				

Chemsex use	Univariate analysis		Multivariate analysis Max model		Multivariate analysis Optimized model	
	OR (95% CI)	*p* value	OR (95% CI)	*p* value	OR (95% CI)	*p* value
HIV	7.08 (2.91–17.25)	<0.05	5.18 (1.50–17.96)	<0.05	6.35 (2.25–17.91)	<0.05
Smoke	2.29 (1.09–4.81)	<0.05	2.44 (0.82–7.30)	0.12	2.76 (1.04–7.32)	<0.05
Alcohol (≥1–2 times/week)	1.29 (0.62–2.64)	0.49	2.02 (0.70–5.84)	0.19	–	
STI history	6.56 (2.18–19.80)	<0.05	1.66 (0.40–6.89)	0.48	–	
Group sex on the last year (≥3 people)	13.31 (5.66–31–31)	<0.05	13.53 (5.34–34.31)	<0.05	13.53 (5.34–34.31)	<0.05
MSM (ref. MSW)	6.83 (1.87–24.95)	<0.05	2.02 (0.44–9.30)	0.37	–	
Provide sexual services	5.28 (0.99–28.3)	<0.05	1.47 (0.10–21.06)	0.78	–	
Pay for sexual services	0.78 (0.15–4.19)	0.77				
Stable partner	0.34 (0.16–0.72)	<0.05				
Condom use	0.55 (0.27–1.13)	0.10				
Anxiety scale	0.95 (0.88–1.03)	0.25				
Depression scale	1.01 (0.92–1.12)	0.78				

STI, sexually transmitted infections; MSM, men who have sex with men; MSW, men who have sex with women; WSM, women who have sex with men; OR, Odds ratio.

## 4. Discussion

In this descriptive study, we found that drug use and chemsex practices were highly prevalent between a population attending an STI clinic. Subjects reporting drug use and chemsex were more frequently cis-men, identified as MSM, living with HIV and with a history of multiple partners or group sex. The ratio of MSM who participated in our survey closely matches the ratio of MSM attending the clinic (according to the statistics of our service, 60% of the people are MSM), and the high prevalence of chemsex detected in this group (49.3%) is slightly higher than described so far in our environment (15). Previous research in MSM living with HIV has reported a chemsex prevalence of 29.1% (16), and data from a study in 13 European cities showed a prevalence of sex-related drug consumption of 30% between MSM, including alcohol and erectile dysfunction medications (17).

Recent data have contributed to the knowledge of this issue in other settings. A French study including 680 university students and using an anonymous online questionnaire similar to ours, found a prevalence of chemsex use of 22.5%, lower compared to the prevalence in our study (33.8%). The most significant associated factors were the use of dating applications, chemsex use with a partner, pornography use, and bisexual orientation (18). Another questionnaire-based study conducted in an STI clinic in Italy found a prevalence of sex-related use of 24.7% during the last year, with a significant association between chemsex consumption and number of previous partners (>5), use of dating apps, reduced condom use and use of PEP between 96 MSM participants (19). The higher prevalence observed in our sample could be explained by the previous association between chemsex use and STIs, as all of our participants were evaluated for an STI diagnosis.

Additionally, in our study, we have found differences in drug consumption patterns according to the type of sexual partner. Other studies have also shown that among MSM, substances like poppers, mephedrone, and GHB/L (20) are the most commonly used, in consonance with our results. Among MSW, we found that cocaine and poppers were the most commonly used drugs after cannabis and alcohol, but the survey did not collect their use in the sexual context. We consider this a relevant limitation given the previous data on this question. In an online survey conducted in Canada (21) to explore how people feel when combining cannabis and sexual activity, most of the respondents used cannabis regularly and reported that when using during sex they were able to increase the intensity of their sensations. Among the differences between men and women, 50% of the women found it easier to reach orgasm compared to 31.4% of the men.

The use of drugs during sex can have detrimental effects on sexual health. It impairs the individual ability to negotiate safer sex practices, leading to a higher number of sexual partners and prolonged and intense sexual sessions (11, 15). Among MSM, participation in recent multipartner chemsex has been associated with a higher risk of syphilis, gonorrhea and chlamydia diagnosis (22), and the prevalence of sex-related drug consumption observed in our sample highlights the association between chemsex and STIs, consistent with previous studies (23–25).

Moreover, engaging in chemsex can significantly negatively affect mental and social well-being. Our survey results indicate that the participants had moderate levels of anxiety. Although we did not find differences between drug users in the HADS score, the reported consequences and experiences related to drug use included episodes of anxiety and impaired and dysfunctional sexual experiences, especially after drug discontinuation. A previous cross-sectional study of chemsex between MSM living with HIV using the HADS scale for mental health evaluation found that chemsex was associated with lower odds of depression and not associated with anxiety (26). However, other previous research links chemsex with increased levels of anxiety, depression, social isolation, stigma, violence, and addiction (26, 27). While different types of drugs have various psychological effects, international organizations identify anxiety as one of the main common symptoms (28). On the other hand, many people develop pathological drug use in an attempt to alleviate the symptoms of anxiety or stress, leading to unsatisfactory sexual relations. For such individuals, chemsex serves as an escape route (28, 29).

Our study has some limitations. The descriptive approach provides a snapshot of the participants’ characteristics and does not allow for tracking changes over time or establishing causal relationships. Participants attending STI clinics may have different characteristics and risk behaviors than those who do not seek medical care (30), implying a potential selection bias as the sample may not fully represent the general population engaging in chemsex. The reliance on self-reported behaviors collected through a self-administered interview introduces the possibility of recall bias. In addition, the ambiguity of some questions may lead to misinterpretation or social desirability bias, where subjects may underreport certain behaviors due to stigma associated with chemsex or personal beliefs about drug use and sexual behaviors. The online nature of the survey poses a limitation in terms of response rate and representativeness of the sample, as it may attract individuals with easier access to the internet, potentially excluding other subjects, such as those with lower socioeconomic status (31, 32). The non-response rate in online surveys can also be higher compared to face-to-face interviews or paper-based surveys (32).

One of the major strengths of our study is the different characteristics of the participants, including women, people living with and without HIV infection, and subjects with variable sexual orientations. It contributes to the limited research focusing on individuals engaged in heterosexual or bisexual relationships, which is an important aspect to consider in chemsex studies. This broadens the knowledge of chemsex practices beyond specific populations and provides insights into the impact across diverse groups.

The implications of this study are relevant for the design and implementation of preventive strategies, targeting chemsex practices, and addressing the psychological and social consequences of chemsex in comprehensive interventions. First, it is crucial to raise awareness among individuals about the risks and harms associated with chemsex and provide accessible testing and treatment services for STIs. Creating a supportive and non-judgmental environment within healthcare settings is essential to foster trust and communication between healthcare professionals and patients, thus facilitating early intervention and care. Additionally, collaborative work between sectors and stakeholders to effectively reach individuals engaged in chemsex is crucial, providing integrated care and addressing the multifaceted challenges associated with drug consumption (11, 15, 27). It is essential to take a balanced approach when discussing chemsex. While sex-related drugs can be associated with potential negative outcomes, such as increased risk of STIs, it is equally important to recognize the diverse motivations and experiences of those who engage in them. Many people who consume chemsex engage in chemsex to enhance their sexual experiences and connect with sexual partners (23). There are several studies in this regard conducted in the MSM population and it would be interesting to expand the boundaries of these studies to the general population. A harm reduction-oriented tone is recommended to address chemsex as a public health problem (20).

Our study emphasizes the urgent need for targeted interventions on prevention, education, and drug use support, as well as the relevance of drug consumption evaluation in clinical practice. Information and counseling can enhance individuals’ ability to be aware and minimize risk factors, encourage seeking care, and promote similar actions among their sexual partners. Future research should aim to overcome these limitations, including larger and more diverse populations, as well as evaluate the effectiveness of interventions for harm reduction and prevention in the context of chemsex.

In conclusion, we found a high prevalence of drug use and chemsex practices, which should prompt public health interventions given their potential impact on the sexual and mental health of people attending STI clinics.

## Data availability statement

The raw data supporting the conclusions of this article will be made available by the authors, without undue reservation.

## Ethics statement

The studies involving humans were approved by Ethics Committee of Ramón y Cajal Hospital. The studies were conducted in accordance with the local legislation and institutional requirements. The participants provided their written informed consent to participate in this study. Written informed consent was obtained from the individual(s) for the publication of any potentially identifiable images or data included in this article.

## Author contributions

MR: Conceptualization, Data curation, Methodology, Writing – original draft, Formal analysis. RR: Conceptualization, Data curation, Methodology, Writing – original draft, Formal analysis. JD-Á: Writing – original draft. JM-S: Validation, Writing – review & editing. SS-V: Supervision, Writing – review & editing. SM: Supervision, Writing – review & editing. MS-C: Supervision, Writing – review & editing.
