# Supplementary material for: Prevalence, characteristics, and associated risk factors of drug consumption and chemsex use among individuals attending an STI clinic (EpITs STUDY)
Source: Front Public Health. 2023 Oct 31;11:1285057. doi: 10.3389/fpubh.2023.1285057 (PMC10644726; doi:10.3389/fpubh.2023.1285057)
Supplement: Supplementary file 1 [file Data_Sheet_1.pdf]

SUPPLEMENTARY MATERIAL

Supplementary 1: EpiTS survey variables.

* Gender: Cis Female / Cis Male / Trans Female / Trans Male / I don't identify with any of them
*Age: numerical
* Place of birth:
Spain / Europe / South America / North America / Central America / Asia / Africa / Oceania
* Educational level:
Not completed primary school / Primary school / Secondary school / University Education
* Employment status: Active / Student / Unemployed / Retired / Other
* Monthly income: No income / <500 / 500-1000 / 1000-2000 / >2000
* Cohabitation: Alone / Couple / Parents / Friends-family / Other
* HIV
Year of diagnosis
PrEp knowledge
On ART
Previous PrEp consumption
* Any previous STI
Any in the last year
* Sexual relations with: Men / Women / Trans women / Trans men
* Usual partner
Couple type: closed/open
From when: <6m / 6m-2a / >2a
HIV serology couple: + / - / Unknown
Condom use: Always / Usually / Sometimes / Never / No sex
* Sexual intercourse in the last year: Alone / Couples / Trio / Group (+3)
Group members: 4-6 / 7-10 / 11-20 / +20
* Sexual partners in the last year: 1 / 2 / 3 / 4 / 6-12 / 12-24 / 24-36 / 36-60 / >60
* Condom use in the last year outside a steady partner:
Always / In more than half of the relationships / less than half of the relationships / Never
* Sexual services rendered for money in the last year
* Sex received in exchange for money in the last year
* Smoking: Never smoked / Ex-smoker / Active
No. of cigarettes/day
* Alcohol consumption in the last year: Never / < once a month / 2-3 times a month / 1-2 times a week / 2-3 times a week / daily or almost daily
* Ever use of drugs
Date of last consumption: <15 days / 15-30 days / 1-3 months / 3-12 months / >1 year
* Type of drug consumed and frequency of consumption:
Cannabis / Poppers / GHB-GBL / Cocaine / Mephedrone / Ecstasy / Methamphetamine / Ketamine / Speed / Others (Specify which)
Cocaine/mephedrone/amphetamine/ketamine route of use:
Ingested / Snorted / Smoked / Anal-genital / Injected
Confirm injected administration

Who injects: Myself / Other person / Variable
Shared injection material
Use of selected drugs during sexual intercourse
Drug use only in the couple's environment: YES/NO
Year of onset of drug use in sex
Use of more than one type of drug: YES/NO
Number of drugs combined: 1 - >10
Place of consumption: Home / Bars / Sex clubs / Cruising / Street
Consumption time/sex: <4h / 4-12 / 12-24 / 24-48 / >48h

Consumption-related experiences:
I have enjoyed drug-free sex less than before
Erection problems, premature or delayed ejaculation with drug-free sex
I have stopped having sex without drugs
Nothing
Negative consequences of consumption:
Unpleasant physical sensations under the effect of drugs
Overdose (loss of consciousness)
Anxiety or panic attacks
Irritability or aggressiveness
Paranoias
Suicidal thoughts
Attempted suicide
Nothing

Perceived interferences of drug use:
At work
In social relationships (friends)
At the family level
In the control of my disease
I do not think so
I do not know

* Use of erectile dysfunction pills in the past year
* Sedative/tranquilizer use in the last year
* Consumption of fitness products in the last year: None / Steroids / Protein Supplements / Other
* Use of sex partner web applications in the last year
Frequency of use: Never / Sometimes / Usually / Always
Applications used
* Monthly drug expenditures: 0 / 1-100 / 101-500 / 501-1000 / >1000

Hospital Anxiety and Depression Scale (HADS)

ANXIETY SCALE: Never / Occasionally / Most of the day / Most of the day / Almost all-day
* I feel tense or nervous
* I feel a kind of fear as if something bad is going to happen.
* I have a head full of worries
* I can sit quietly and relaxed.
* I experience an unpleasant feeling of nervousness and tingling in my stomach.

* I feel restless as if I can't stop moving
* I suddenly experience feelings of great distress or fear.

DEPRESSION SCALE: Often / Sometimes / Rarely / Almost never
* I continue to enjoy things as usual
* I can laugh and see the funny side of things.
* I feel cheerful
* I feel slow and clumsy
* I have lost interest in my appearance
* I look forward to things
* I can enjoy a good book or a good radio or TV program.

The cut-off points are as follows: 0-7: normal / 8-10: probable case of anxiety or depression / 11-21: case of anxiety or depression.

**Supplementary 2: Sociodemographic and sexual related characteristics by chemsex use**	
	n=49
Age	37 (27-47)
Genre	47 (96%)
Male cis	0 (0%)
Female cis	0 (0%)
Male trans	0 (0%)
Not identified with previous	0 (0%)
HIV	21 (43%)
Previous PreP use	6 (12%)
Previous STI (last year)	33 (67%)
Sexual partner	
MSM	41 (84%)
MSW	3 (6%)
WSM	0 (0%)
WSW	0 (0%)
Bisexual Men	3 (6%)
Bisexual Women	0 (0%)
Stable partner	
Yes	14 (29%)
No	35 (71%)
Sexual partners in the last year	

1	1 (2%)
2-4	14 (29%)
6-12	8 (16%)
12-24	10 (20%)
24-36	6 (12%)
>36	4 (8%)
Groupal sex in the last year (>3)	31 (63%)
Condom use	
>50% intercouse	24 (49%)
<50% intercourse	24 (49%)
Sexual services provided	5 (10%)
Sexual services hired	2 (4%)
Anxiety scale	7.54 (3.32-11.76)
Depression scale	5.09 (1.62-8.56)

Data are presented as mean (SD) for continuous measures, and n (%) for categorical measures.
Prep: preexposure prophylaxis; STI: sexually transmitted infections; MSM: men who have sex with
men; MSW: men who have sex with women; WSM: women who have sex with men; WSW: women
who have sex with women

**Supplementary 3. Table 4: Consequences and experiences with drug use**	n=96
I have enjoyed drug-free sex less than before	9 (9%)
Erection problems, premature or delayed ejaculation with drug-free sex	10 (10%)
I have stopped having drug-free sex	6 (6%)
Unpleasant physical sensations under the effect of drugs	13 (14%)
Overdose	1 (1%)
Anxiety or panic attacks	7 (7%)
Irritability or aggressiveness	5 (5%)
Paranoia	4 (4%)
Suicidal ideation	2 (2%)
Suicide attempt	0 (0%)
Interference at work	7 (7%)
Interference in social relations	4 (4%)
Family interference	3 (3%)
Interference in health (HIV control)	4 (4%)
